# Real-World Comprehensive Genomic Profiling Success Rates in Tissue and Liquid Prostate Carcinoma Specimens

**DOI:** 10.1093/oncolo/oyac181

**Published:** 2022-09-07

**Authors:** Matthew C Hiemenz, Ryon P Graf, Kelsie Schiavone, Lukas Harries, Geoffrey R Oxnard, Jeffrey S Ross, Richard S P Huang

**Affiliations:** Foundation Medicine, Inc., Cambridge, MA, USA; Foundation Medicine, Inc., Cambridge, MA, USA; Foundation Medicine, Inc., Cambridge, MA, USA; Foundation Medicine, Inc., Cambridge, MA, USA; Foundation Medicine, Inc., Cambridge, MA, USA; Foundation Medicine, Inc., Cambridge, MA, USA; Department of Pathology, Upstate Medical University, Syracuse, NY, USA; Foundation Medicine, Inc., Cambridge, MA, USA

**Keywords:** prostate cancer, precision medicine, real-world

## Abstract

Challenges with sequencing tissue samples from patients with prostate cancer have been reported in clinical trials. To assess the success rate of comprehensive genomic profiling (CGP) for prostate cancer patients, we analyzed a real-world cohort who underwent sequencing of their prostate tissue sample as well as a subset of patients with a reflex liquid biopsy. Overall, a significant majority (82%) of tissue prostate carcinoma samples yielded reportable CGP results. Of those samples that were unsuccessful, most (75%) were inadequate samples that did not meet pre-established criteria to advance into sequencing. For cases where liquid CGP was performed if tissue CGP was unsuccessful, mutations that were likely attributable to prostate carcinoma were observed in most cases and all cases were successful in generating a report. These results suggest that, for CGP testing, prostate cancer tissue is a reasonable matrix type and that liquid samples can be effectively used as an alternative to tissue.

## Teaser

To assess the success rate of comprehensive genomic profiling for patients with prostate cancer, a real-world cohort that underwent sequencing of prostate tissue samples was analyzed, including a subset of patients with a reflex liquid biopsy.

## Introduction

Current guidelines recommend tumor testing for homologous recombination repair (HRR) gene alterations in patients with metastatic prostate cancer.^1^ While comprehensive genomic profiling (CGP) can capture the full range of HRR gene alterations, sample inadequacy and sub-optimal CGP success rates in tissue specimens have been identified as an issue in clinical trials (eg, PROfound trial).^2,3^ We hypothesized that (1) due to the use of archival samples and extensive specimen partitioning for biomarker correlate analyses in clinical trials, the success rate of tissue CGP in standard clinical practice might be higher and (2) among patients with inadequate or unsuitable specimens for tissue testing, relevant genomic alterations might be identified with reflex liquid biopsy.

## Methods

**Figure 1.**
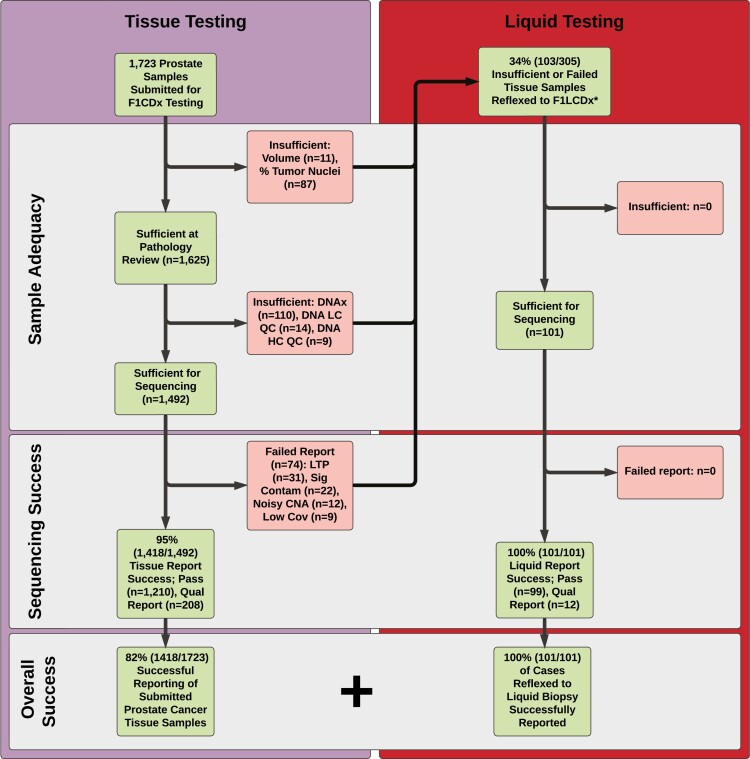
Real world prostate carcinoma testing success rate by processing step and assay.

We analyzed all prostate cancer tissue specimens submitted for CGP testing between October 1, 2020 and December 31, 2020. CGP of tissue samples was performed with the FDA-approved FoundationOne CDx assay using previously described methods.^4^ For tissue samples that initially did not generate a successful report (i.e., insufficient or failed samples), repeat testing was possible in some circumstances (depending on the availability of residual tumor material). When repeat testing was not possible, the clinician who initially ordered the testing could elect to send in an additional tissue specimen or, alternatively, provide a liquid sample. Of the specimens where tissue CGP testing did not result in a successful report, we queried our database to see if a subsequent FDA-approved liquid CGP test (FoundationOne Liquid CDx) was ordered.^5^ Causes for insufficient or failed tissue samples are listed in Fig. 1.

*Of the 103 specimens reflexed to F1LCDx, there were two patients who each submitted two tissue samples; both samples from each of these two patients did not generate a successful report. This means that while there was 103 tissue CGP specimen reflexed to F1LCDx, there were only 101 patients and hence only 101 F1LCDx ordered from the 103 tissue specimens. For tissue specimens that did not yield a successful report, causes include insufficient tissue volume, insufficient % tumor nuclei, insufficient DNA extraction yield, library construction quality control not met, hybrid capture quality control not met, low tumor purity in the absence of reportable variants, significant contamination, excessive copy number noise, and low coverage.

Abbreviations: F1CDX, FoundationOne CDx; NGS, next-generation sequencing; DNAx, DNA extraction yield; LC, library construction; QC, quality control; HC, hybrid capture; F1LCDX, FoundationOne Liquid CDx; Qual, qualified (qualified reports carry a disclaimer that the sensitivity may be reduced to one or more factors related to sample quality); LTP, low tumor purity; Sig Contam, significant contamination; Noisy CNA, noisy copy number alteration data; Low cov, low coverage.

## Results

Of all samples received, 12.1% (208/1723) were inadequate due to insufficient tissue volume, insufficient % tumor nuclei, or insufficient DNA yield prior to sequencing (Fig. 1). In addition, 1.3% (23/1723) of samples submitted for testing did not pass the quality control metrics for library construction or hybrid capture prior to sequencing and 4.3% of successfully sequenced samples generated a failed report (74/1723). Therefore, 13.4% of samples were inadequate for testing whereas 4.3% of samples failed following the sequencing process.

In one-third of inadequate or failed tissue samples (33.8%, 103/305), the ordering provider chose to order liquid CGP as a reflex test for the unsuccessful tissue CGP. 100% (101/101) of reflexed liquid biopsy samples generated a report with sequencing results. For the cases that were either successfully sequenced tissue samples or failed tissue samples that were reflexed to liquid CGP, 100% (1519/1519) led to a clinical report with sequencing results.

Analysis of the reflexed liquid biopsy samples in this study revealed variants in genes commonly mutated in prostate cancer including *TP53* (43.2%), *AR* (28.8%), *TMPRSS2* (14.4%), *CDK12* (9.0%), *BRCA2* (2.7%), and *PTEN* (13.5%).

## Discussion

The results of this study suggest that the real-world success rate for CGP testing of prostate cancer tissue specimens with an FDA-approved assay is significantly higher than what has been reported in a large clinical trial.^3^ This finding is likely attributable to differences between real-world samples and clinical trial samples which are often archival and subject to another ancillary testing before genomic profiling. A combined sample inadequacy and failure rate of 42.5% was reported in phase III of the PROfound trial.^3^ In this study, only 17.7% of real-world tissue samples did not successfully generate a report and the majority (75.7%) were inadequate samples that did not meet pre-established criteria to advance into sequencing. Of note, inadequate samples include exhausted tissue blocks and specimens without tumors. For tissue site, 60.9% of samples were primary prostate specimens whereas 39.1% were in a metastatic site (Supplementary Table S1). For sample age, the mean time from collection to pathology review was 683 days while the median time was 195 days (Supplemental Table S2). For the PROfound trial, 82.8% of samples were primary prostates and 16.6% were metastatic (0.6% were site unknown).^3^ The mean sample age for all samples in this trial was 1732 days.^3^ Examining HRR mutations, 351 patients out of 1723 patients screened had an HRR mutation (20.4%); in the PROfound trial, 19.2% of patients screened had an HRR mutation.^6^ A comparison of the incidence of specific reportable HRR mutations identified in HRR(+) patients to the enrolled patients with eligible HRR mutation in the PROfound study is listed in Supplemental Table S3.

A subset of the real-world tissue samples that did not generate a successful CGP report were reflexed to liquid CGP testing. For these reflexed liquid biopsy samples, 100% were successful in generating a report (Fig. 1). Of note, we cannot exclude the possibility that some of the liquid biopsies may have been ordered on the basis of a higher likelihood of obtaining a result (ie, widely metastatic or advanced disease). Two phase III clinical trials, PROpel and MAGNITUDE, are currently examining the utility of combining a PARP inhibitor with an androgen pathway inhibitor in patients with metastatic, castration-resistant prostate cancer.^7,8^ Both trials are assessing HRR mutations using both tissue and liquid samples, highlighting the complementary nature of these two approaches.

The limitations of this study include the aggregate comparison of tissue success rates, the possibility of false negative results from liquid biopsy due to insufficient tumor shedding, and the smaller sample size for the liquid biopsy cohort.

These data support prostate cancer tissue samples being a reasonable matrix type for CGP testing in routine clinical specimens. Our data additionally suggest that liquid CGP is an acceptable alternative when prostate tissue CGP does not result in a successful report, for molecular profiling at the time of recurrence, or when a new concurrent CGP analysis is needed to search for a resistance mechanism to the most recently prescribed anti-tumor therapy.

## Supplementary Material

oyac181_suppl_Supplementary_Tables

## Data Availability

The data underlying this article will be shared on reasonable request to the corresponding author.
