# Supplementary material for: Real-World Comprehensive Genomic Profiling Success Rates in Tissue and Liquid Prostate Carcinoma Specimens
Source: Oncologist. 2022 Sep 7;27(12):e970–2. doi: 10.1093/oncolo/oyac181 (PMC9732218; doi:10.1093/oncolo/oyac181)
Supplement: oyac181_suppl_Supplementary_Tables [file oyac181_suppl_supplementary_tables.docx]

**Table S1. Sample characteristics**

| Specimen Site | Number of Samples | % of Samples |
| --- | --- | --- |
| **Primary:** | 1049 | 60.9% |
| Prostate | 1049 | 60.9% |
| **Metastatic:** | 674 | 39.1% |
| Abdomen | 4 | 0.2% |
| Adrenal Gland | 3 | 0.2% |
| Bladder | 61 | 3.5% |
| Bone | 140 | 8.1% |
| Brain | 3 | 0.2% |
| Chest Wall | 1 | 0.1% |
| Colon | 2 | 0.1% |
| GEJ | 1 | 0.1% |
| Head and Neck | 4 | 0.2% |
| Liver | 87 | 5.0% |
| Lung | 40 | 2.3% |
| Lymph Node | 196 | 11.4% |
| Mediastinum | 2 | 0.1% |
| Omentum | 2 | 0.1% |
| Pelvis | 19 | 1.1% |
| Peritoneum | 4 | 0.2% |
| Peritoneal Fluid | 1 | 0.1% |
| Pleura | 2 | 0.1% |
| Pleural Fluid | 1 | 0.1% |
| Rectum | 8 | 0.5% |
| Retroperitoneum | 18 | 1.0% |
| Soft Tissue | 28 | 1.6% |
| Spine | 43 | 2.5% |
| Testis | 2 | 0.1% |
| Trachea | 1 | 0.1% |
| Urethra | 1 | 0.1% |

**Table S2. Time from collection date to pathology review.**

|  | Collection Date to Pathology Review Date (Days) |
| --- | --- |
| mean | 683 |
| median | 195 |
| range | 1 to 7064 |

**Table S3. Presence of Homologous Recombination Repair (HRR) Mutations in HRR(+) Cohort. Patients with multiple genes are included across more than one gene.**

| Gene | % Patients in HRR(+) Cohort (Current Study) with Gene Mutation (n = 351) | % Enrolled Patients with Eligible HRR Mutation in PROfound Study (n = 387) |
| --- | --- | --- |
| *BRCA1* | 3.7% | 3.9% |
| *BRCA2* | 36.8% | 37.5% |
| *ATM* | 22.5% | 23.8% |
| *BRIP1* | 1.7% | 1.0% |
| *BARD1* | 2.0% | 1.0% |
| *CDK12* | 20.2% | 25.6% |
| *CHEK1* | 0.9% | 0.8% |
| *CHEK2* | 7.1% | 4.4% |
| *FANCL* | 0.6% | 0.0% |
| *PALB2* | 4.0% | 2.1% |
| *PPP2R2A* | 3.4% | 3.9% |
| *RAD51B* | 1.7% | 1.8% |
| *RAD51C* | 1.7% | 0.0% |
| *RAD51D* | 1.4% | 0.5% |
| *RAD54L* | 1.7% | 1.6% |
